# Handmade Patient-Specific Bolus Combined With Photon Radiation Therapy for Skin Cancer

**DOI:** 10.1155/crom/5598014

**Published:** 2025-06-10

**Authors:** Mostafa Robatjazi, Sajedeh Sadat Jamalabadi, Sajjad Beynabaji, Hamid Reza Baghani, Seyed Alireza Javadinia

**Affiliations:** ^1^Non-Communicable Diseases Research Center, Sabzevar University of Medical Sciences, Sabzevar, Iran; ^2^Department of Medical Physics and Radiological Sciences, Sabzevar University of Medical Sciences, Sabzevar, Iran; ^3^Student Research Committee, Sabzevar University of Medical Sciences, Sabzevar, Iran; ^4^Department of Physics, Hakim Sabzevari University, Sabzevar, Iran

**Keywords:** BCC, bolus, photon, radiation therapy, SCC, skin cancer

## Abstract

**Introduction:** Skin cancer is the most prevalent cancer in Iran. While surgical excision is the primary treatment, radiation therapy (RT) plays a crucial role, especially for tumors in critical anatomical locations. This case report evaluates the performance of handmade boluses for skin cancer RT using 6 MV photons.

**Case Presentation:** Two patients with lip squamous cell carcinoma (SCC) and nasal basal cell carcinoma (BCC) were treated using 6 MV photon RT with handmade boluses. Dosimetric evaluation using EBT2 Gafchromic film was performed to verify the delivered radiation doses. For the lip SCC patient, the measured dose was 3.9% higher than the planned 73.85 Gy. For the nasal BCC patient, the measured dose was 3.48% higher than the planned 75.60 Gy, demonstrating the high accuracy of the handmade bolus approach.

**Discussion:** The use of patient-specific, handmade boluses demonstrated several advantages, including reduced air gaps and improved dose delivery accuracy compared to commercial boluses. The consistent bolus positioning minimized interfraction setup variations, leading to lower standard deviations in the measured doses. While this study did not directly quantify the air gap, prior research has reported air gap reductions of over 72% with patient-specific boluses.

**Conclusion:** This case report supports the effectiveness of handmade boluses in combination with 6 MV photons for skin cancer RT, particularly in resource-constrained settings where advanced treatment modalities may not be readily available. The handmade bolus approach provides an accessible solution to enhance the precision of skin cancer RT.

## 1. Introduction

Skin cancer is the most prevalent form of cancer in Iran, with epidemiological data indicating it as the leading cancer type for men (standardized incidence rate of 18.93) and the second most common cancer affecting women (standardized incidence rate of 13.09) in the country [1, 2]. Over 95% of all skin cancer diagnoses among these malignancies are basal cell carcinoma (BCC) and squamous cell carcinoma (SCC) [3]. These statistics underscore the significant burden of skin cancer within the Iranian population and the importance of developing effective treatment strategies to address this public health concern. Surgical excision, cryotherapy, radiation therapy (RT), and topical agents are various treatment options for these malignancies [3, 4]. While surgical excision is considered the primary curative treatment approach for BCC and cSCC, in some critical locations, it may induce cosmetic defects or functional disorders. RT plays an important role in both definitive and adjuvant settings in these situations.

While electron beams are predominantly used to treat skin tumors, they can be associated with certain drawbacks from a physics perspective. One of the main issues is the potential for an inhomogeneous dose distribution, and, in some cases, it can lead to inadequate target coverage, especially on irregular surfaces [5, 6]. Photon beams can also be employed as an alternative method, but the use of a bolus is necessary to improve the effectiveness of RT and mitigate the skin-sparing effect, which is known as the build-up effect. The bolus is used to augment the dose and improve the coverage of superficial tumors [5–7]. Nevertheless, the use of conventional boluses presents some challenges in RT. Failures in the conformal contact of the bolus with irregular skin surfaces, such as those found on the ear, nose, and scalp of the patient, lead to inadequate dose coverage and dose homogeneity because of the flaky structure of conventional boluses [6, 8–10].

Some studies showed that the use of a three-dimensional (3D) bolus may offer several advantages over conventional boluses in RT. These studies have reported that patients treated with a 3D bolus experienced significantly lower rates of complications compared to those treated with a conventional bolus. The superior conformity and reduced air gaps provided by 3D boluses appear to be a key factor in minimizing the risk of complications for patients undergoing RT. [6, 10, 11] These findings suggest that the use of 3D bolus technology may offer clinical benefits compared to conventional bolus approaches. The 3D boluses can be prepared using either 3D printing or in handmade format. While 3D printing is not available widely in all of the radiotherapy facilities, handmade boluses can be considered a valid option. This study sought to evaluate the performance of handmade boluses prepared using a thermoplastic material in the treatment of two patients with lip and nose SCC using 6 MV photon RT.

## 2. Case Presentation

**Figure 1:**
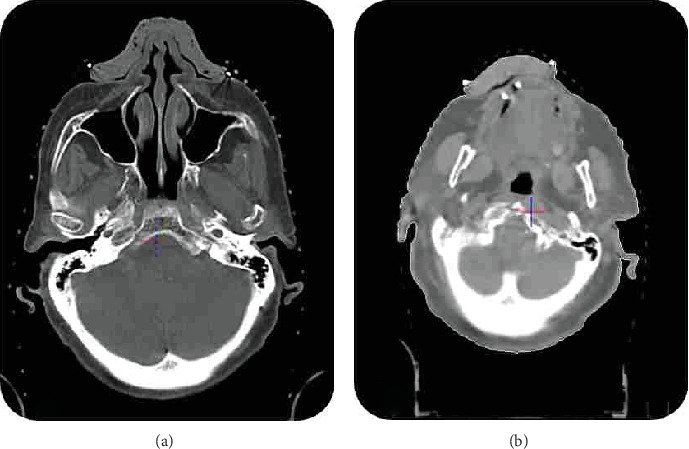
CT images of presented cases with handmade bolus on target. (a) Bolus on the nose of the patient during CT simulation. (b) Bolus on the lip of the patient during CT simulation.

Two patients with skin cancer were referred to our department for definitive radiotherapy using a 6 MV linear accelerator. The first patient, an 84-year-old female, presented with SCC of the lower lip. The second, a 67-year-old female, had BCC of the nose. Both required RT to the affected lip and nose, respectively, with a prescribed dose of 70 Gy delivered in 35 fractions. The irregular skin surfaces of these anatomical sites necessitated a customized, handmade bolus to optimize radiation dose delivery by eliminating the skin-sparing effect of 6 MV photons. The bolus was constructed using low-melting-point polycaprolactone-based thermoplastic granules (melting point about 60°C). The granules were heated in a water bath at 65°C–70°C for 5–10 min until pliable, stirred to remove air bubbles, and molded directly onto a nonstick silicone mold based on pretreatment CT images (1–2 mm slice thickness). The bolus thickness was standardized at 5–10 mm, determined by treatment planning to achieve 90%–100% surface dose, and verified using a caliper at multiple points (variation < ±0.8 mm). To ensure homogeneity and minimize air gaps, the material was pressed gently to conform to the contours of the lip or nose. After cooling in a cold-water bath (10°C–15°C) for 2–3 min, the bolus was trimmed and inspected for defects. To ensure there was no significant air gap between the bolus and the patient's skin, CT images were reviewed slice by slice by both a radiation oncologist and a medical physicist. This thorough visual inspection helped confirm adequate contact between the bolus and skin surface in both cases. CT imaging verified bolus fit and uniformity (Figure 1).

The handmade bolus was carefully positioned on the patient's skin surface, and the EBT2 Gafchromic film was placed between the bolus and the patient's skin. This allowed for the direct measurement of the radiation dose delivered through the bolus and onto the target area. The calibration curve and corresponding equation relating the optical density of the EBT2 Gafchromic film to the absorbed radiation dose, which was obtained in our previously published study, was utilized in the current study [12].

Due to the lack of an electron beam mode in the RT equipment available at our institution, located in a developing country, 6 MV photon beams were utilized for the treatment of the patients in this study. The use of photon beams, rather than electron beams, necessitated the use of bolus to improve the dose distribution and coverage of the superficial tumors located on the lip and nose.

Following the treatment delivery, the exposed films were scanned and analyzed using the recommended protocol to quantify the dose distribution under the bolus region. The measured doses were then compared to the planned dose distribution to verify the effect of the handmade bolus in delivering the intended RT.

The percentage difference between the planned and measured doses was calculated using Equation (1).

(1) $$\begin{array}{c} \text{Percentage difference}=\frac{\text{Planned dose}‐\text{Measured dose}}{\text{Planned dose}}*100. \end{array}$$

This dosimetric evaluation provided valuable insight into the performance of the handmade bolus and its ability to conform to the irregular anatomy of the lip and nose, ensuring optimal dose coverage to the target volumes while minimizing the risk of hot or cold spots.

The radiation doses delivered to the target volumes were measured and compared to the planned doses using EBT2 Gafchromic film placed between the handmade bolus and the patients' skin surfaces in three fractions.

For the patient with lip SCC, the planned dose to the surface of the target was 73.85 Gy delivered in 35 fractions. Analysis of the exposed Gafchromic films revealed that the actually delivered dose to this area was 76.73 Gy. This represented a difference of only 3.9% more than the planned dose.

 $$\begin{array}{c} \text{Percentage Difference}=\frac{73.85\text{ Gy}–76.73\text{ Gy}}{73.85\text{ Gy}}\times 100\%=3.90\%. \end{array}$$

Similarly, for the patient with nasal BCC, the planned dose on the surface of the target was also 75.60 Gy in 35 fractions. Dosimetric evaluation using the Gafchromic film showed that the measured dose on this area was 78.23 Gy, a difference of 3.48% more than the planned dose.

 $$\begin{array}{c} \text{Percentage Difference}=\frac{75.60\text{ Gy}–78.23\text{ Gy}}{78.23\text{ Gy}}\times 100\%=3.48\%. \end{array}$$

These results demonstrate the high degree of accuracy achieved with the handmade bolus in delivering the intended radiation doses to the superficial lip and nasal skin cancer targets. The negligible differences between the planned and measured doses highlight the effectiveness of the customized bolus approach in ensuring optimal dose coverage to the treatment areas.

## 3. Discussion

In this study, we demonstrated the effectiveness of handmade boluses for skin cancer RT using 6 MV photons. We employed EBT2 film dosimetry to verify dose delivery and evaluate the performance of patient-specific boluses with 6 MV photons. Our findings indicate that combining 6 MV photons with patient-specific boluses offers an effective treatment strategy, particularly in developing countries, where advanced RT technologies, such as electron beams or brachytherapy systems, may not be widely accessible. This approach proves valuable for treating skin cancers in complex anatomical regions, including the lip, nose, and other irregular areas.

The application of a patient-specific bolus reduced the air gap between the treatment surface and the bolus material, as illustrated in Figure 1, thereby enhancing the accuracy of the delivered radiation dose. Compared to a commercial bolus, the patient-specific bolus exhibited superior dose delivery precision, especially in regions with larger air gaps. This improvement likely stems from the challenges associated with maintaining consistent positioning of the commercial bolus across multiple treatment fractions. The commercial bolus's position and fit were more prone to interfraction setup variations, resulting in inconsistent air gaps and diminished dose accuracy. The presence of airgaps can lead to dose underestimation at the skin surface and near-surface regions due to the lack of electronic equilibrium. This is particularly critical in treatments involving superficial targets. Strategies to minimize this effect include careful bolus placement, custom-molded bolus materials, and verification using imaging applicable for this purpose. Conversely, the patient-specific bolus used in this study maintained consistent positioning, even in the presence of an air gap. This reliability in placement minimized the effects of interfraction setup variations, as evidenced by the reduced standard deviations in measured dose values when the patient-specific bolus was employed. Although our study did not directly measure or compare air gaps, prior research underscores the advantages of patient-specific boluses in this regard. For instance, Wang et al. and Kim et al. [11, 13] reported air gap reductions exceeding 72% with patient-specific boluses compared to commercial alternatives, a finding consistent with Kim et al. [14], who utilized a life-casting method to achieve a 77.62% reduction. The ability to stabilize the air gap through customized boluses appears critical to enhancing dose delivery precision in skin cancer RT.

Further supporting these observations, Wang et al. [11] conducted in vivo dosimetry comparisons between standard and handmade boluses. Their results revealed that a standard bolus produced a mean absolute difference of 5.69 ± 4.56% (maximum 15.1%) between expected and measured radiation doses, whereas the handmade bolus achieved a significantly lower mean absolute difference of 1.91 ± 1.31% (maximum 3.51%). Our study's findings align with those of Wang et al., confirming that our handmade bolus performs comparably to customized boluses in their research. A key advantage of our approach lies in its reliance on a handmade, custom-fabricated bolus rather than 3D-printed ones, making it more adaptable to healthcare settings lacking the infrastructure or resources for 3D printing technology.

Moreover, recent studies have explored advanced techniques for patient-specific radiotherapy devices. Kim et al. [14] demonstrated significant air gap reductions using a life-casting method, while Jreije et al. [15] developed 3D-printed devices that improved dose verification accuracy. Similarly, Asfia et al. [16] reviewed 3D-printed immobilization devices, noting their potential but emphasizing the need for additional validation. In contrast, our study utilized a handmade bolus crafted from thermoplastic granules and verified doses with EBT2 film, offering a practical, cost-effective alternative that requires no specialized equipment. This accessibility enhances its suitability for resource-limited environments, distinguishing it from methods reliant on advanced technologies.

The effectiveness of the EBT2 film-based verification approach was previously demonstrated in our earlier work [12], where we demonstrated that EBT2 film dosimetry beneath conventional boluses in postmastectomy RT yielded approximately a 4% difference from the planned dose. Additionally, our earlier studies confirmed that EBT2 film exerts no significant perturbation effect on the delivered dose, reinforcing its utility in this context [17, 18].

Although we ensured uniform bolus thickness during the construction process, one potential limitation of our study was the challenge of maintaining this uniformity across the entire bolus structure, which may introduce minor dosimetric uncertainties. However, these variations are likely negligible, given the consistent dose accuracy observed in our measurements.

## 4. Conclusion

This case report demonstrates the effectiveness of using handmade boluses in combination with 6 MV photons for skin cancer RT. Reduction in the air gap and improved dose delivery accuracy are the technical keys in the use of patient-specific boluses.

Overall, the findings from this case report support the use of patient-specific boluses as an effective and accessible solution to enhance the precision of skin cancer RT, especially in developing countries with limited access to advanced treatment modalities.

## Data Availability

The data that support the findings of this study are available from the corresponding author upon reasonable request.
